# Mitochondrial Dysfunction as a Therapeutic Target in COPD

**Published:** 2017

**Authors:** Kian Fan CHUNG

**Affiliations:** National Heart & Lung Institute, Imperial College, London UK

Mitochondria are key regulators of metabolism, redox homeostasis, and cell survival and proliferation. They are the most important source of intracellular reactive oxygen species generated during the synthesis of ATP through oxidative phosphorylation, and mitochondrial reactive oxygen species (mtROS) are key regulators of mitochondrial function. Failure to scavenge mtROS leads to mitochondrial dysfunction as shown in airway epithelial cells exposed to CSE leading to decreased ATP levels and mitochondrial membrane potential and impaired mitophagy. In addition, mitochondrial fragmentation, branching and reduced cristae are linked to increased levels of IL-6, CXCL8 and IL-1β and to senescence features.

Airway smooth muscle cells (ASMCs) cultured from patients with COPD have a reduced basal respiration rate, reduced ATP-linked respiration, a reduced reserve capacity for oxidative phosphorylation and increased proton leak, that were associated with reduced expression of Complex I, III and V, enhanced mtROS, and decreased membrane potential and ATP production. Exogenous hydrogen peroxide reduced intracellular ATP levels in ASMCs from all groups but only COPD ASMCs showed increased mtROS levels, accompanied by the greatest increase in IL-6, CXCL8 and GM-CSF expression.Mitochondrial dysfunction in the murine ozone-exposed lung cells as reflected by decreased membrane potential, increased mtROS and reduced mitochondrial complex protein I, III & V expression. Isolated mitochondria from bronchial biopsies of COPD patients had a lower membrane potential and increased mtROS compared to healthy non-smokers, with a close correlation between membrane potential and FEV1, transfer factor to carbon monoxide and peak oxygen consumption.

Mito-Q10, a derivative of ubiquinone conjugated to triphenylphosphonium, a lipophilic cation that allows entry into the mitochondriuminhibited proliferation of ASMCs from patients with COPD with reversal of mitochondrial dysfunction leading to reduced inflammation and airway hyperresponsiveness after acute exposure to ozone in a mouse model of COPD. Co-culture of induced pluripotent stem-cell derived mesenchymal cells (iPSC-MSC) with ASMCs led to the protection of ASMCs from mtROS-induced deterioration of membrane potential and apoptosis when cells were exposed to CSE, achieved through the transfer of mitochondria from iPSC-MSC cells. Similarly, in an in-vivo oxidative stress model of single ozone exposure, intravenously administrated iPSC-MSCs were able to prophylactically reduce ozone-induced airway hyperresponsiveness, airway inflammation, apoptosis, mtROS and reduction of membrane potential while the therapeutic administration of iPSC-MSCs reduced the ozone-induced apoptosis and mtROS.

iPSC-MSCs and mitochondrial-targeted antioxidants are effective in protecting against oxidative stress-induced mitochondrial dysfunction in the lungs and are promising therapies for COPD.

